# Building a global alliance of biofoundries

**DOI:** 10.1038/s41467-019-10079-2

**Published:** 2019-05-09

**Authors:** Nathan Hillson, Mark Caddick, Yizhi Cai, Jose A. Carrasco, Matthew Wook Chang, Natalie C. Curach, David J. Bell, Rosalind Le Feuvre, Douglas C. Friedman, Xiongfei Fu, Nicholas D. Gold, Markus J. Herrgård, Maciej B. Holowko, James R. Johnson, Richard A. Johnson, Jay D. Keasling, Richard I. Kitney, Akihiko Kondo, Chenli Liu, Vincent J. J. Martin, Filippo Menolascina, Chiaki Ogino, Nicola J. Patron, Marilene Pavan, Chueh Loo Poh, Isak S. Pretorius, Susan J. Rosser, Nigel S. Scrutton, Marko Storch, Hille Tekotte, Evelyn Travnik, Claudia E. Vickers, Wen Shan Yew, Yingjin Yuan, Huimin Zhao, Paul S. Freemont

**Affiliations:** 1 DOE Agile BioFoundry, Emeryville, CA 94608 USA; 2 GeneMill, Institute of Integrative Biology, University of Liverpool, Liverpool, L69 7ZB UK; 3 SYNBIOCHEM, Manchester Institute of Biotechnology and School of Chemistry, University of Manchester, Manchester, M13 9PL UK; 4 Earlham Institute, Norwich Research Park, Norfolk, NR4 7UZ UK; 5 NUS Synthetic Biology for Clinical and Technological Innovation (SynCTI), Department of Biochemistry, Yong Loo Lin School of Medicine, National University of Singapore, Singapore, 117456 Singapore; 6 Bioplatforms Australia, Research Park Drive, Macquarie University, Macquarie Park, NSW 2109 Australia; 7 London DNA Foundry, Imperial College Translation & Innovation Hub, White City Campus, 80 Wood Lane, London, W12 0BZ UK; 8 Engineering Biology Research Consortium (EBRC), Emeryville, CA 94608 USA; 9 Shenzhen Institute of Synthetic Biology, Shenzhen Institutes of Advanced Technology, Chinese Academy of Sciences, Shenzhen, People’s Republic of China; 10 Centre for Applied Synthetic Biology, Concordia University, Montreal, Montreal, QC H4B 1R6 Canada; 11 The Novo Nordisk Foundation Center for Biosustainability, Technical University of Denmark, 2800 Kongens Lyngby, Denmark; 12 CSIRO Synthetic Biology Future Science Platform, Canberra, ACT 2601 Australia; 13 Australian Institute for Bioengineering and Nanotechnology, The University of Queensland, Brisbane, QLD 4072 Australia; 14 Department of Molecular Sciences, Macquarie University, Macquarie, NSW 2109 Australia; 15 Global Helix LLC, BioBricks Foundation, and Engineering Biology Research Consortium (EBRC), Emeryville, CA 94608 USA; 16 Graduate School of Science, Technology, and Innovation, Kobe University, Kobe, 657-8501 Japan; 17 UK Centre for Mammalian Synthetic Biology SynthSys, School of Biological Sciences, University of Edinburgh, Edinburgh, EH93FF UK; 18 DAMP Lab, Biological Design Center, Boston University, Boston, MA 02215 USA; 19 Macquarie University, North Ryde, NSW 2109 Australia; 20 Frontier Science Center for Synthetic Biology (MOE), Tianjin University, Tianjin, People’s Republic of China; 21 Illinois Biological Foundry for Advanced Biomanufacturing (iBioFAB), University of Illinois at Urbana-Champaign, Urbana, IL 61801 USA

**Keywords:** Metabolic engineering, Synthetic biology

## Abstract

Biofoundries provide an integrated infrastructure to enable the rapid design, construction, and testing of genetically reprogrammed organisms for biotechnology applications and research. Many biofoundries are being built and a Global Biofoundry Alliance has recently been established to coordinate activities worldwide.

Over the past 5 years, research institutions around the world have been establishing biofoundries to expand their biotechnology development capacities. However, the existence of these biofoundries is not yet widely known within the biotechnology or broader biological research communities. Biofoundries aim to accelerate and enhance both academic and translational research in engineering/synthetic biology by promoting and enabling the beneficial use of automation and high-throughput equipment including process scale-up, computer-aided design software, and other new workflows and tools. Iterative Design-Build-Test-Learn biological engineering cycles (Fig. 1) allow researchers to test large-scale genetic designs and apply artificial intelligence (AI)/machine learning to enhance the design process. Other goals include building a robust engineering/synthetic biology industry as well as accelerating the commercialization of engineering/synthetic biology and biomanufacturing process engineering. One aspirational goal is to establish biodesign rules that can be applied for the efficient reprogramming of living cells for biotechnology and biomedical applications. Such reprogramming will also allow fundamental insights into the complexity of living systems. In terms of throughput, current exemplars include the Edinburgh Genome Foundry^1^, which for example can process over 2000 DNA assembly reactions per week, 20-times the throughput of a single person without automation. At the University of Illinois at Urbana-Champaign, iBioFAB^2^ can build up to 1000 TALEN constructs per day at <$3 each, 0.3% of what it might otherwise cost. Moreover, iBioFAB can perform multiplex genome-scale engineering of *Saccharomyces ceresvisiae* in a fully automated manner^3^, >10-times the throughput of a single person without automation. Working with small companies, the London DNA Foundry^4^, Singapore SynCTI Foundry^5^ and US DOE Agile BioFoundry^6^ now provide cost-effective access to expensive equipment and the necessary expertize for product prototyping and commercial process validation, which are often required to secure additional capital investment.

**Fig. 1:**
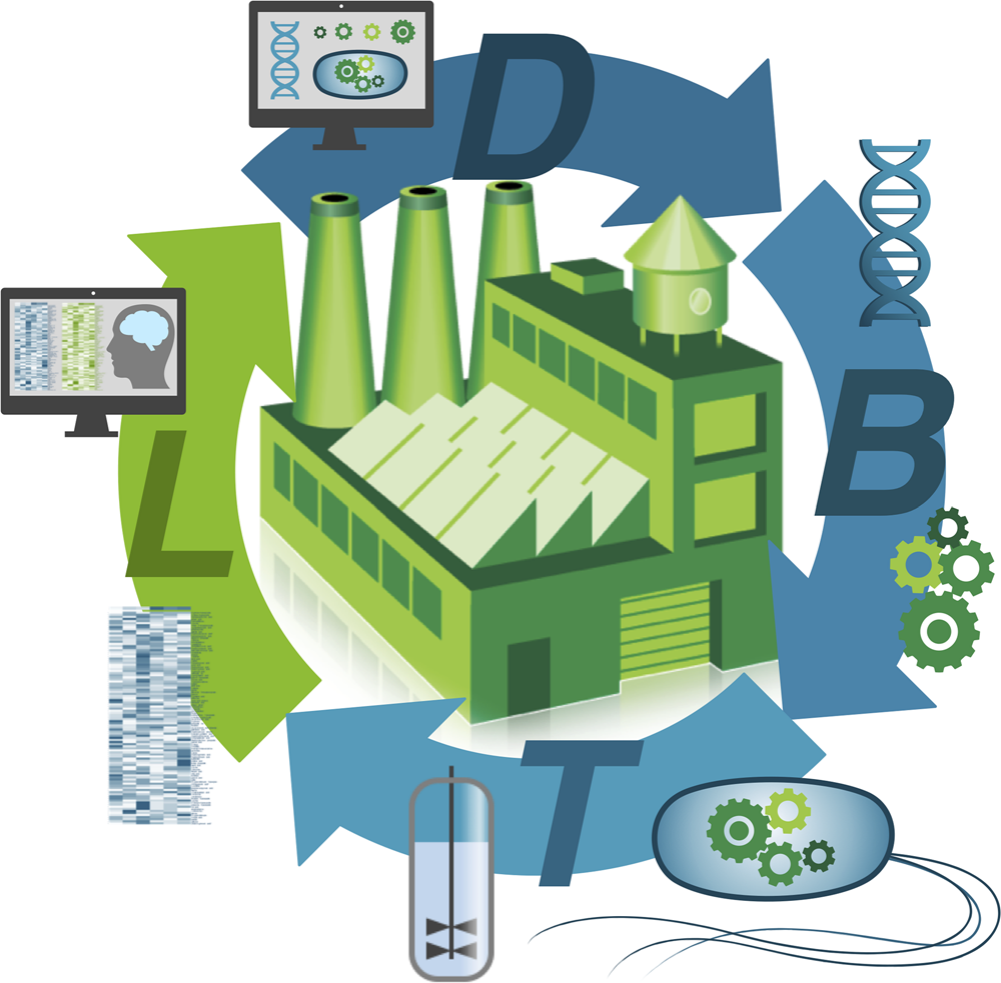
The Design-Build-Test-Learn (DBTL) biological engineering cycle. In simple terms the DBTL framework aims to fulfill particular design criteria for a synthetic biology application, which might for example be the production of a specific product at an optimal titer or the detection of a specific clinical biomarker using an engineered gut microbiome. The cycle begins with D (Design), which defines the desired target function/specifications and involves the computational design of genetic parts, circuits, regulatory and metabolic pathways to whole genomes; B (Build) involves the physical assembly of those designed genetic components; T (Test) involves the prototyping and testing of the assembled genetic designs in living cells (also called "chasses") at different scales, which also includes comprehensive analytical measurements (‘‘omics’’) of specific cellular components. This can also include testing components in cell-free extract systems; L (Learn) is the application of modeling and computational learning tools, which uses the data obtained in T to inform the design process. Iterations of the DBTL cycle results in genetic designs that aim to fulfill the design specifications established in D. In the figure the DBTL cycle is depicted around an imagined biofactory or biorefinery where many products will be produced using more sustainable and circular economic processes forming the future infrastructure for a global bioeconomy. (Credit: Christopher Johnson, DOE Agile BioFoundry, Golden, CO, USA)

To enable global coordination of these efforts, 15 non-commercial biofoundries from four continents gathered in London in June 2018 to discuss the formation of a Global Biofoundry Alliance (GBA) that would enable the collective to share experiences and resources and work together to overcome shared challenges and unmet scientific and engineering needs. Several operational challenges reverberated through the June meeting. Participants frequently expressed concerns over sustainable biofoundry growth and development, commenting on the high costs of retaining staff, maintaining infrastructure, and replacing equipment at a predictable rate. Small user bases and a limited awareness of biofoundry capabilities among potential users currently limit the revenue streams that could eventually offset these expenditures. High experimental costs are also a significant barrier for the academic community and indicate that an extended period of public investment will be necessary to enable the benefits of biofoundries to impact the broader research community. Standards were also a key challenge with scant software, hardware, and methodological standardization in place to promote interoperability, efficiency and safety. Legal issues associated with sharing intellectual property, physical samples, and other resources across national and institutional boundaries have also proven difficult. Using BioFoundries effectively requires a paradigm shift in how we do biological engineering, and training the next generation to effectively exploit these technologies is also extremely important. Notwithstanding these challenges, it was agreed that biofoundry capabilities are a critical enabling technology for individual countries to develop capability and deliver on the significant promise of synthetic/engineering biology.

The GBA will be formally launched on 9th May, 2019 in Kobe, Japan, during a meeting of the Founding Members (Fig. 2). The GBA has agreed to a non-binding Memorandum of Understanding, which does not establish any legal rights or obligations, but is a voluntary arrangement dependent upon goodwill and cooperation. Signing parties are research institutions, research funding agencies, or other entities that operate non-commercial biofoundries, as well as other organizations that actively support public-funded biofoundries. The parties have non-overlapping missions with for-profit entities. The objectives of the GBA are to

1. Develop, promote, and support non-commercial biofoundries established around the world,
2. Intensify collaboration and communication among biofoundries,
3. Collectively develop responses to technological, operational, and other types of common challenges,
4. Enhance visibility, impact and sustainability of non-commercial biofoundries, and
5. Explore globally relevant and societally impactful grand challenge collaborative projects.

**Fig. 2:**
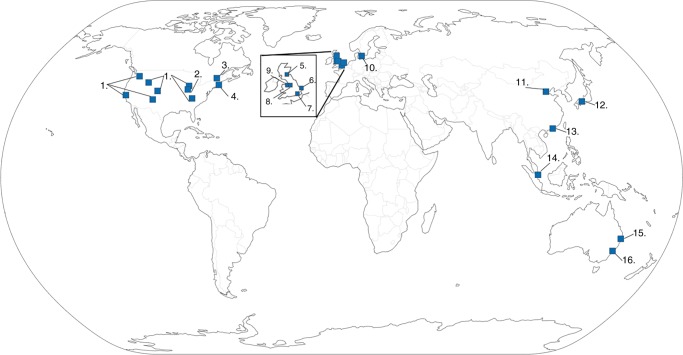
Map of the Global Biofoundry Alliance. Map of the world showing the geographical locations of founding members of the Global Biofoundry Alliance with each biofoundry numbered. (1) DOE Agile BioFoundry (pending) located across Emeryville, CA, Richland, WA, Golden, CO, Lemont, IL, Los Alamos, NM, Oak Ridge, TN, and Idaho Falls, ID sites; (2) Illinois Biological Foundry for Advanced Biomanufacturing (iBioFAB), University of Illinois at Urbana-Champaign; (3) Concordia Genome Foundry, Concordia University Montreal; (4) DAMP lab, Boston University; (5) Edinburgh Genome Foundry, University of Edinburgh; (6) Earlham Institute, Norwich Research Park; (7) London DNA Foundry, Imperial College London; (8) SYNBIOCHEM, University of Manchester; (9) GeneMill University of Liverpool; (10) Novo Nordisk Foundation Center for Biosustainability, Technical University of Denmark; (11) Frontier Science Center for Synthetic Biology (MOE), Tianjin University; (12) Graduate School of Science, Technology and Innovation, Kobe University; (13) Shenzhen Institute of Synthetic Biology, Shenzhen Institutes of Advanced Technology, Chinese Academy of Sciences; (14) NUS Synthetic Biology for Clinical and Technological Innovation (SynCTI), National University of Singapore; (15) Australian Foundry for Advanced Biomanufacturing (AusFAB), University of Queensland and (16) Australian Genome Foundry, Macquarie University. The Plotly.Py library was used to create the graphic and is open source under MIT license (https://github.com/plotly/plotly.py/blob/master/LICENSE.txt)

To achieve these objectives, the GBA will provide coordination between Alliance members and promote collective action and sharing of pre-competitive infrastructure, open standards, protocols, best practices, bio-parts, and data where possible. This will also involve exploring standardized legal tools to reduce the transaction costs of sharing including the OpenMTA^7^. The GBA will also allow increased visibility about the role and importance of biofoundries by reporting on success stories and positive impacts. Other activities will involve the exchange of sustainable business models, as well as approaches to lowering transaction and operational costs and expanding user bases; and personnel exchanges, including developing teaching and training programs for researchers and users of biofoundry facilities. The GBA will actively and transparently engage a broad range of stakeholders including policy makers, industry, public funding and government agencies, as well as civil society to continually improve GBA activities and practices.

To strengthen the coordination and collaboration within the GBA, the Alliance will also explore opportunities to tackle a globally relevant, societally impactful grand challenge (e.g., directly addressing one of the UN sustainable development goals) with each biofoundry bringing its unique strengths and capabilities to the problem. GBA members will also explore bilateral collaborations on smaller-scale projects (e.g., biofoundry performance testing and benchmarking). The benefits of the GBA are anticipated to be analogous to those experienced by the synthetic yeast genome Sc2.0^8^ project, in which internationally distributed participant teams share a common scientific and engineering biodesign goals.
