# Author Correction: Building a global alliance of biofoundries

**DOI:** 10.1038/s41467-019-10862-1

**Published:** 2019-07-11

**Authors:** Nathan Hillson, Mark Caddick, Yizhi Cai, Jose A. Carrasco, Matthew Wook Chang, Natalie C. Curach, David J. Bell, Rosalind Le Feuvre, Douglas C. Friedman, Xiongfei Fu, Nicholas D. Gold, Markus J. Herrgård, Maciej B. Holowko, James R. Johnson, Richard A. Johnson, Jay D. Keasling, Richard I. Kitney, Akihiko Kondo, Chenli Liu, Vincent J. J. Martin, Filippo Menolascina, Chiaki Ogino, Nicola J. Patron, Marilene Pavan, Chueh Loo Poh, Isak S. Pretorius, Susan J. Rosser, Nigel S. Scrutton, Marko Storch, Hille Tekotte, Evelyn Travnik, Claudia E. Vickers, Wen Shan Yew, Yingjin Yuan, Huimin Zhao, Paul S. Freemont

**Affiliations:** 1 DOE Agile BioFoundry, Emeryville, CA 94608 USA; 2 GeneMill, Institute of Integrative Biology, University of Liverpool, Liverpool, L69 7ZB UK; 3 SYNBIOCHEM, Manchester Institute of Biotechnology and School of Chemistry, University of Manchester, Manchester, M13 9PL UK; 4 Earlham Institute, Norwich Research Park, Norfolk, NR4 7UZ UK; 5 NUS Synthetic Biology for Clinical and Technological Innovation (SynCTI), Department of Biochemistry, Yong Loo Lin School of Medicine, National University of Singapore, Singapore, 117456 Singapore; 6 Bioplatforms Australia, Research Park Drive, Macquarie University, Macquarie Park, NSW 2109 Australia; 7 London DNA Foundry, Imperial College Translation & Innovation Hub, White City Campus, 80 WoodLane, London, W12 0BZ UK; 8 Engineering Biology Research Consortium (EBRC), Emeryville, CA 94608 USA; 9 Shenzhen Institute of Synthetic Biology, Shenzhen Institutes of Advanced Technology, Chinese Academy of Sciences, Shenzhen, People’s Republic of China; 10 Centre for Applied Synthetic Biology, Concordia University, Montreal, Montreal, QC H4B 1R6 Canada; 11 The Novo Nordisk Foundation Center for Biosustainability, Technical University of Denmark, 2800 Kongens Lyngby, Denmark; 12 CSIRO Synthetic Biology Future Science Platform, Canberra, ACT 2601 Australia; 13 Australian Institute for Bioengineering and Nanotechnology, The University of Queensland, Brisbane, QLD 4072 Australia; 14 Department of Molecular Sciences, Macquarie University, Macquarie, NSW 2109 Australia; 15 Global Helix LLC, BioBricks Foundation, and Engineering Biology Research Consortium (EBRC), Emeryville, CA 94608 USA; 16 Graduate School of Science, Technology, and Innovation, Kobe University, Kobe, 657-8501 Japan; 17 UK Centre for Mammalian Synthetic Biology SynthSys, School of Biological Sciences, University of Edinburgh, Edinburgh, EH93FF UK; 18 DAMP Lab, Biological Design Center, Boston University, Boston, MA 02215 USA; 19 Macquarie University, North Ryde, NSW 2109 Australia; 20 Frontier Science Center for Synthetic Biology (MOE), TianjinUniversity, Tianjin, People’s Republic of China; 21 Illinois Biological Foundry for Advanced Biomanufacturing (iBioFAB), University of Illinois at Urbana-Champaign, Urbana, IL 61801 USA

**Keywords:** Metabolic engineering, Synthetic biology

Correction to: *Nature Communications* 10.1038/s41467-019-10079-2, published online 9 May 2019.

The original version of this Comment contained errors in the legend of Figure 2, in which the locations of the fifteenth and sixteenth GBA members were incorrectly given as ‘(15) Australian Genome Foundry, Macquarie University; (16) Australian Foundry for Advanced Biomanufacturing, University of Queensland’. The correct version replaces this with ‘(15) Australian Foundry for Advanced Biomanufacturing (AusFAB), University of Queensland and (16) Australian Genome Foundry, Macquarie University’. This has been corrected in both the PDF and HTML versions of the Comment.

